# Targeting IL-13 as a Host-Directed Therapy Against Ulcerative Colitis

**DOI:** 10.3389/fcimb.2018.00395

**Published:** 2018-11-06

**Authors:** J. Claire Hoving

**Affiliations:** Institute of Infectious Disease and Molecular Medicine, Department of Pathology, Faculty of Health Sciences, University of Cape Town, Cape Town, South Africa

**Keywords:** ulcerative colitis, interleukin-13, IL-4 receptor-alpha, drug targets, Inflammatory Bowel Disease, T-helper type 2 immune response

## Abstract

The role of interleukin-13 in mediating ulcerative colitis remains under scrutiny. Compelling evidence from both man and mouse suggests that IL-13 not only contributes to the pathology associated with disease but is also involved in mediating the inflammatory response. These studies have led to the approach of targeting IL-13 as a promising treatment strategy in alleviating ulcerative colitis disease. Despite this evidence, recent clinical trial data suggests that specifically blocking the receptor through which IL-13 signals, IL-4 receptor-alpha (IL-4Rα) in ulcerative colitis patients, is insufficient in protecting them from disease outcome. This challenges the importance of IL-13 as a therapeutic target. This review describes the role of IL-13 in ulcerative colitis and current treatment strategies that target IL-13. The potential role of IL-13 signaling independently of IL-4Rα in mediating ulcerative colitis is highlighted as an important consideration when targeting the signaling mechanisms of IL-13 for therapeutic approaches.

## Introduction

Since the discovery of IL-13 it has been shown to be a key cytokine in controlling pathogens such as helminthic parasites, but also as a prominent feature in allergic and inflammatory diseases. IL-13 is a 10–14 kDa immune-regulatory cytokine first described as a protein preferentially produced by activated T helper-Type (Th) 2 cells (Brown et al., 1989; McKenzie et al., 1993; Minty et al., 1993; Hershey, 2003). However, it has since been established that IL-13 is in-fact produced by a wide variety of cell types, including innate immune cells, with diverse biological activities (Wynn, 2003; Mannon and Reinisch, 2012). These include basophils, eosinophils, mast cells, natural killer cells, epithelial cells, smooth muscle cells, fibroblasts, and NK T cells. This highlights the role of cells other than T and B cells that produce IL-13; however the role of these cells specifically in the gut is yet to be established. Once produced IL-13 initiates a cascade of immunological process which aid in parasite clearance. Here IL-13 can act directly on macrophages, driving the differentiation toward the M2 phenotype resulting in a Th2 response (Gordon, 2003). Furthermore, IL-13 drives beneficial responses such as, the IgE isotype switch, eosinophil recruitment, mucus production and muscle contraction. However, these are the very responses that contribute to pathology in an inflammatory response. Our own studies have shown that blocking IgE in an animal model of ulcerative colitis reduces the severity of disease (Hoving et al., 2012). This was associated with reduced mast cell activation as described below. The pleiotropic nature of IL-13 quickly became evident by the myriad of diseases in which it plays either a beneficial or detrimental role. IL-13 is a prominent mediator of allergic lung disease, including pulmonary inflammation, asthma and anaphylaxis (Gour and Wills-Karp, 2015). More recently, IL-13 has been linked to enhancing brain function in mice by increasing cognitive memory (Brombacher et al., 2017). Here, IL-13 was able to stimulate primary astrocytes to produce brain-derived neurotrophic factor, known to enhance cognitive function. Furthermore, a new SNP in the regulatory region of *il13* (rs1881457C) has been associated with an increased risk of Coronary Artery Disease in a Chinese Han co-hort (Zha et al., 2018). Here the functional mechanisms remain unknown.

## IL-13 and its role in colitis

**Figure 1:**
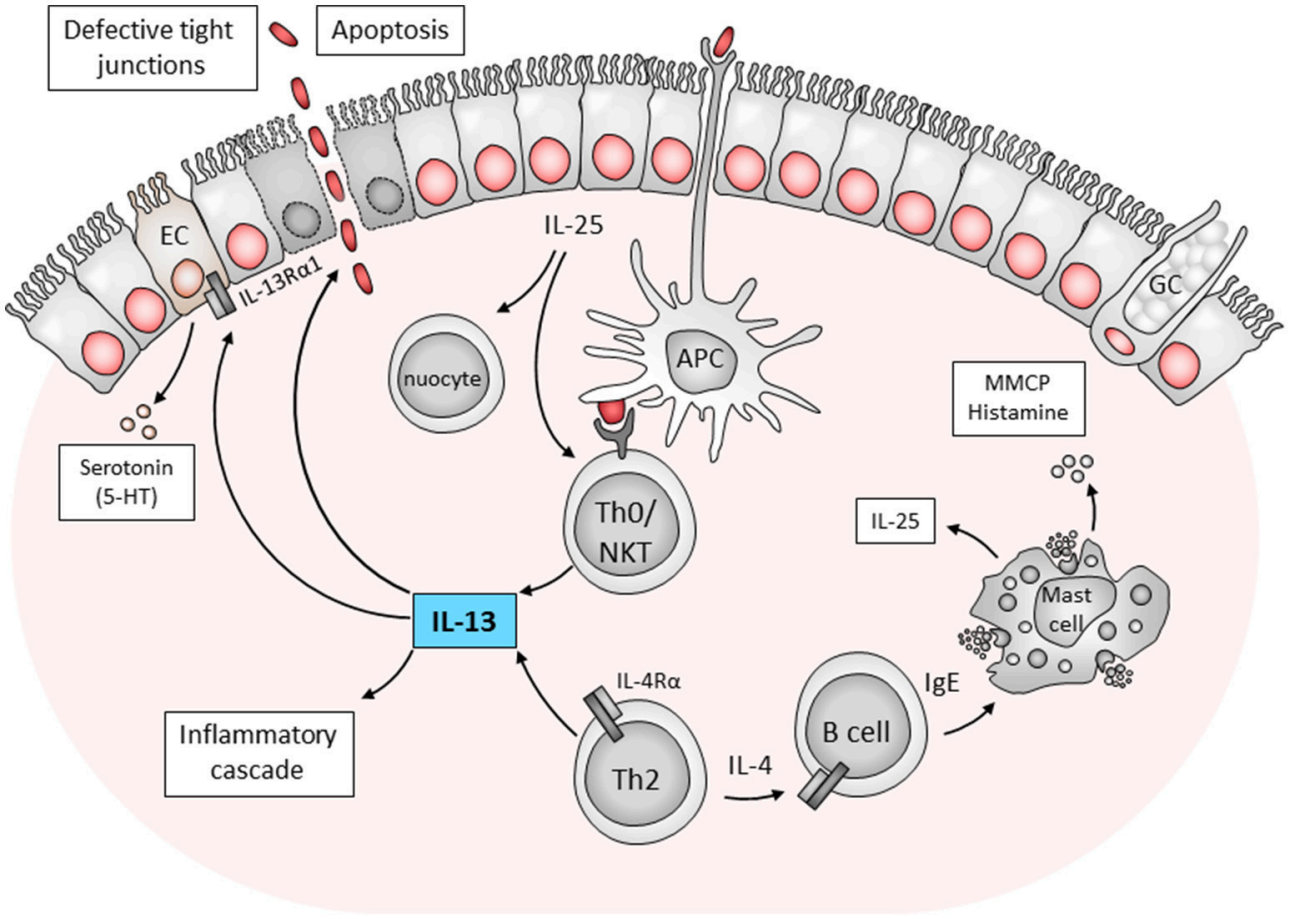
Immune components contributing to ulcerative colitis. The mechanisms of pathogenesis is postulated as follows; a defect in antigen sampling by antigen presenting cells (APCs) or direct stimulation from epithelial cells activates Th0 cells or NK T cells to drive a Th2/type 2 response. Here IL-25 production by epithelial cells was also linked to disease pathology through nuocytes and NK T cells. NK T cells produce IL-13 which is toxic to epithelial cells. Furthermore, conventional CD4+ Th2 cells which produce IL-4 can also stimulate B cells to drive inflammation in an IgE-dependent manner. These findings indicate the complex interaction of host cells in the development of ulcerative colitis. APC, antigen presenting cell; EC, enterochromaffin cell; GC, goblet cell; IL, interleukin; MMCP, murine mast cell protease; NKT, natural killer T cell; Th0, T helper type; 5-HT- serotonin 5-hydroxytryptamine (Heller et al., 2002; Ikeda et al., 2003; Ghia et al., 2009; Camelo et al., 2012; Hoving et al., 2012).

IL-13 is an interesting cytokine in the role it plays in mediating Th2 inflammatory diseases. Initially IL-13 was a target for host directed therapy for asthma, dermatitis and other allergic diseases. However, IL-13 has also been linked to mediating the host inflammatory cascade responsible for the pathogenesis of ulcerative colitis. Combining evidence from mice and man, the mechanism of IL-13 mediated colitis is illustrated in Figure 1. Essentially, a defect in defect in antigen recognition triggers an inappropriate and exaggerated immune response. This is further aggravated by the disruption of epithelial tight junctions, increasing permeability of the intestinal epithelium and resulting in an increase in the uptake of luminal antigens. In a mouse model of ulcerative colitis using the hapten, oxazolone to induce a transient disease phenotype, blocking IL-13 (Heller et al., 2002) or using IL-13 gene-deficient mice (Weigmann et al., 2008) has been shown to ameliorate or prevent disease induction. While IL-13 production by NK T cells has been shown to play a major role in mediating disease, our own studies have implicated additional components of the immune response that contribute to the onset and maintenance of disease. These include IL-4Rα-responsive CD4^+^ T cells and IgE production by B cells which contribute to oxazolone-induced pathology in mice. Depleting IgE was linked to a reduction in the number of activated mast cells and reduced pathology (Hoving et al., 2012).

Increasing evidence demonstrates that IL-13 is responsible for initiating the detrimental inflammatory cascade in colitis. While orchestrating an inflammatory response by immune cells IL-13 can also act directly on epithelial cells. In ulcerative colitis, IL-13 has been described as a key effector cytokine acting on epithelial cell function and initiating apoptosis (Heller et al., 2005, 2008). The addition of IL-13 *in vitro* to HT-29 epithelial cell monolayers causes an increased expression of the pore-forming tight junction protein claudin-2 (Rosen et al., 2011). The increased expression of this protein was associated with increased epithelial barrier permeability. As a consequence, small antigens enter the gut and the loss of ions and water into the intestinal lumen leads to diarrhea. Independent to the role on claudin-2, IL-13 was recently shown to downregulate tricellulin expression. Tricellulin is a protein essential for the barrier against macromolecules and is reduced in ulcerative colitis but not Crohn's disease (Krug et al., 2017). While IL-13Rα1 upregulates claudin-2 in ulcerative colitis, IL-13Rα2 downregulates tricellulin, allowing macromolecule uptake.

Additional studies have expanded on the current understanding of the role IL-13 plays in colitis and describe additional mechanisms associated with IL-13 during colitis. For example, in the oxazolone colitis mouse model, blocking IL-25 derived from intestinal epithelial cells improved the clinical aspects of disease (Camelo et al., 2012). This was associated with reduced IL-13 production by lamina propria cells, fewer NKT cells, and nuocytes infiltrating the mucosa and a decrease in serum IgE levels. Interestingly, mast cells have previously been shown to be potent producers of IL-25 (Ikeda et al., 2003), which could in turn also contribute to the downstream immunological cascade seen in ulcerative colitis. Therefore, not only could IL-25 be involved in initiating disease, but also maintaining the detrimental Th2 response in established disease. Interestingly, in the Dextran sulfate sodium (DSS) hapten-induced mouse model of ulcerative colitis, serotonin production by enterochromaffin cells of the mucosa was implicated in disease (Ghia et al., 2009). More recently, this serotonin production was linked to IL-13, and highlights the interaction between the immune-endocrine axis in IL-13-mediated gut inflammation (Shajib et al., 2013). These mechanistic insights into disease pathogenesis could provide additional host directed IL-13 drug targets to alleviate the symptoms of ulcerative colitis.

## IL-13 signaling mechanisms

**Figure 2:**
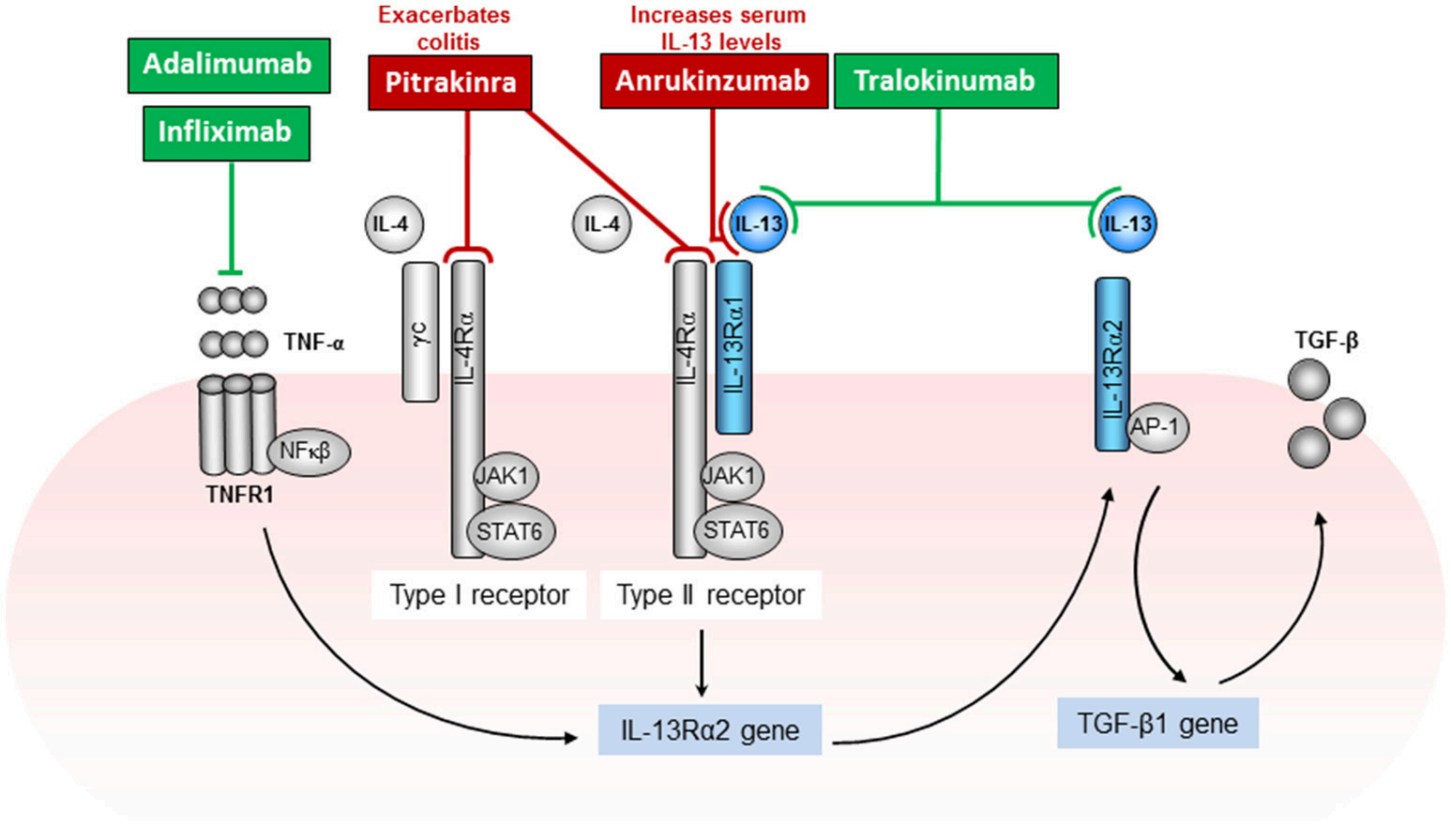
IL-13 signaling mechanisms and associated host directed targets in the treatment of ulcerative colitis. IL-13 signals through the type II (IL-4Rα and IL-13Rα1) receptor complex and activates the JAK1/STAT6 pathway. In addition, IL-13 has also been shown to signal through IL-13Rα2, activating AP-1 to induce the secretion of TGF-β. This pathway is, in part dependent on the production of TNFα. Various drug targets have been directed at IL-13 including the IL-4Rα signaling pathway to block the immune response that mediate Th2-driven inflammatory diseases such as allergy and colitis. Drug treatments that were beneficial or improved disease outcome are depicted in green and treatments that were unsuccessful or exacerbated disease outcome are depicted in red (Rutgeerts et al., 2005; Wenzel et al., 2007; Levin and Shibolet, 2008; Reinisch et al., 2011, 2015; Mannon and Reinisch, 2012; Sandborn et al., 2012; Verma et al., 2013; Colombel et al., 2014; Feagan et al., 2014; Danese et al., 2015; Palamides et al., 2016; Hoving et al., 2017; Popovic et al., 2017).

Both IL-4 and IL-13 cytokines use the IL-4Rα chain as a component of their receptors (Figure 2). This was shown in mice treated with anti-IL-4Rα antibodies or IL-4 antagonists (Aversa et al., 1993), which specifically blocked responses of both IL-4 and IL-13 (Zurawski et al., 1993, 1995; Hilton et al., 1996). The IL-4Rα consists of a 140-kDa IL-4Rα chain which is a component of both the type I and type II IL-4 receptors. The IL-4Rα is expressed in relatively low numbers on numerous cell types. The type I IL-4 receptor results from association of IL-4Rα with the gamma common (γc) chain, which is also a component of the receptors for IL-2, IL-7, IL-9, and IL-15 (McKenzie et al., 1993). The type II IL-4/IL13 receptor results from association of IL-4Rα with IL-13Rα1. The type II receptor is composed of the IL-4Rα chain and the 65-70 kDa IL-13Rα1 chain and serves as an alternative receptor for IL-4 (Figure 2). By itself, IL-13Rα1 binds IL-13 with low affinity but when paired with IL-4Rα, it binds IL-13 with high affinity and forms a functional unit that signals (McKenzie et al., 1993; Miloux et al., 1997). IL-13Rα1 is expressed on the majority of cell types tested with the exception of human or mouse T cells (Hershey, 2003). Although IL-13 signals via the IL-13Rα1, it has a higher binding affinity to the α2 chain of the IL-13 receptor (IL-13Rα2), which has previously been considered as a decoy receptor for IL-13 with no signal transduction. IL-13Rα2 is a 55–60 kDa protein closely related to IL-13Rα1 except that the cytoplasmic domain has no signaling motifs or binding sequences for signaling molecules (Donaldson et al., 1998). However, more recent publications have highlighted a possible signaling pathway for IL-13 through the IL-13Rα2. IL-13 signaling through the IL-13Rα2 was shown to be involved in the induction of TGF-β1 production or mediating fibrosis in a chronic mouse model of Crohn's disease (Fichtner-Feigl et al., 2006, 2008).

IL-4 and IL-13 not only share common subunits, they also share common signaling pathways (Figure 2). The components of both receptor complexes are associated with Janus kinases (JAK). JAK1 has been proposed to associate with the IL-4Rα chain, γc with JAK3 and IL-13Rα1 with JAK2 (Nelms et al., 1999). The signal transducer and activator of transcription 6 (STAT6) is recruited to the phosphorylated IL-4Rα where it also becomes phosphorylated by JAKs (Nelms et al., 1999). Studies using STAT6-deficient mice have determined that IL-13 signaling uses the JAK/STAT6 pathway (Takeda et al., 1996). In fact, in the oxazolone colitis model in which an increased epithelial cell, T cell, macrophage, and NKT cell STAT6 phosphorylation was observed, STAT6-deficient mice demonstrated a reduced disease phenotype (Rosen et al., 2013). Until recently the IL-13Rα2, which binds IL-13 with high affinity, was thought to relay no signal. Fichtner-Feigl and colleagues have shown that IL-13 signaling through IL-13RαI/IL-4Rα together with TNF-α signaling through TNFR1, up regulates IL-13Rα2 surface expression on macrophages. IL-13 binding this receptor activates AP-1 to induce the secretion of TGF-β (Fichtner-Feigl et al., 2006).

## Host directed treatment strategies against IL-13

Considering the compelling link between IL-13 and ulcerative colitis, various clinical trials have been implemented to target IL-13 as a treatment strategy. A previous review by Mannon and Reinisch elegantly summarized drug targets for IL-13 in the treatment of colitis, however at the time most of these trials were ongoing and the outcomes unknown (Mannon and Reinisch, 2012). Conflicting results from recent clinical trials taint the optimism for using anti-IL-13 treatments for ulcerative colitis. However, a better understanding of the signaling mechanism of IL-13 and associated drug target sites could provide a useful approach for treatment strategies. Anrukinzumab (IMA-638) is a humanized monoclonal antibody which binds IL-13 and prevents the cytokine from binding IL-4Rα but maintains the ability to bind both IL-13Rα1 and IL-13Rα2 (Figure 2). In a multicenter, randomized, double-blind, placebo-controlled study, patients with active UC received anrukinzumab or placebo treatment (Reinisch et al., 2015). The primary endpoint was fold change from baseline in fecal calprotectin, a protein released into the intestine and recognized as a marker for active inflammatory bowel disease. IL-13 levels increased in treated patients, and no improvement was reported. Considering that IL-13 would still be able to bind other IL-13 receptors, this trial indicates the potential of IL-13 to mediate colitis independently of the IL-4Rα. In fact the mean fecal calprotectin levels in the patients receiving the highest dose of 600 mg anrukinzumab was actually increased at week 14. The authors conclude that there was no significant therapeutic effect of anrukinzumab on patients with active UC and that the study had a high drop-out rate due to the lack of efficacy.

Another recent candidate in ulcerative colitis treatment is tralokinumab, a human IL-13-neutralizing IgG4 monoclonal antibody. This monoclonal antibody has a very high affinity for IL-13 and is at stage III clinical trials for both allergic asthma and atopic dermatitis. A recent study characterizing the structure of tralokinumab Fab in complex with IL-13 demonstrates the inhibition of binding to both IL-13Rα1 and IL-13Rα2 (Popovic et al., 2017). Analyzing the structure in detail defined the mechanism of interactions and demonstrated that tralokinumab inhibits the formation of the tertiary complex among IL-13, IL-13Rα1, and IL-4Rα and inhibits the complex formation between IL-13 and IL-13Rα2. In a randomized, double-blind, placebo-controlled, phase IIa study for the treatment of ulcerative colitis, tralokinumab did not significantly improve the clinical response (Danese et al., 2015). These results were discouraging; however patients did have a higher clinical remission rate and improved mean partial Mayo score. Furthermore, patients in the study were not classified according to their base-line mucosal IL-13 levels. Considering that UC patients have been shown to have differential expression profiling of downstream inflammatory cytokines depending on the severity of disease (Verma et al., 2013), there may be the potential to identify specific patient groups with increased IL-13 as a biomarker for which tralokinumab treatment would be most beneficial.

Many treatment approaches against ulcerative colitis target the IL-4Rα signaling pathway. This is based on the fact that both IL-4 and IL-13 signal through the IL-4Rα. However, in light of recent studies describing the potential role of IL-13Rα2 in IL-13 signaling, this treatment approach may not be appropriate in ulcerative colitis (Fichtner-Feigl et al., 2006, 2008). Pitrakinra is a recombinant human IL-4 protein, (rather than a monoclonal antibody such as anrukinzumab and tralokinumab) which is mutated and therefore prohibits complex formation between IL-4Rα and IL-2Rγ or IL-13Rα1, but has no known effect on IL-13Rα2 (Wenzel et al., 2007). In a promising study of asthma, two phase IIa clinical trials demonstrated improved control over asthma symptoms after treatment with Pitrakinra (Wenzel et al., 2007). Based on this success, Pitrakinra treatment was used in a mouse model of ulcerative colitis (Palamides et al., 2016). To overcome some of the caveats of using chemically-induced animal models of colitis, the authors described a new model of ulcerative colitis. Essentially, NOD-scid IL2Rγnull mice reconstituted with peripheral blood mononuclear cells derived from UC-affected individuals develop colitis-like symptoms when challenged with ethanol. In this model pitrakinra showed no therapeutic benefit. In fact, treatment was associated with exacerbated symptoms and pathological manifestations. This outcome supports our own data from the oxazolone mouse model in which mice deficient of IL-4Rα presented with significantly exacerbated disease phenotype (Hoving et al., 2017). Here, mice that do not produce IL-13 (IL-4Ra/IL-13-deficient mice) are protected from colitis. However, the disease phenotype in the adoptive transfer model mentioned above was not directly associated with IL-13 and it would be interesting to know the outcome of pitrakinra treatment using the oxazolone colitis model. Thus, attempts to block the IL-4Rα signaling pathway may actually exacerbate disease outcome.

Lastly, anti-TNFα has proven to be effective in patients that do not respond to convention treatment strategies. In fact this therapeutic approach was the first approved for inflammatory bowel disease treatment more than 50 years ago. As TNFα production is traditionally associated with a Th1 response, it is therefore not a likely candidate for ulcerative colitis treatment. None-the-less, monoclonal antibodies against TNFα such as infliximab and adalimumab have shown promising outcomes (Rutgeerts et al., 2005; Levin and Shibolet, 2008; Reinisch et al., 2011; Sandborn et al., 2012; Colombel et al., 2014; Feagan et al., 2014). Studies describing the effect of anti-TNFα treatment on patient IL-13 production are very limited. It can be postulated that the success of anti-TNFα therapy in ulcerative colitis could be linked to the mechanism behind IL-13Rα2 signaling (Figure 2). Fichtner-Feigl and colleagues, elegantly describe the signaling pathway during the activation of the IL-13Rα2 (Figure 2). Here IL-13Rα2 gene expression was shown to be dependent on TNFα and could provide, at least in part, a link between the IL-4Rα-independent signaling of IL-13 and successful anti-TNFα treatment in ulcerative colitis patients (Fichtner-Feigl et al., 2006).

## Conclusion

The potential benefits of ameliorating IL-13 production in ulcerative colitis remains an interesting approach in treating disease. Furthermore, these treatment strategies could potentially be extended beyond the pathology of colitis. Targeting IL-13 in allergic diseases and dermatitis has already proven to be promising. If the optimal treatment strategy and correct targets can be identified for ulcerative colitis, this could even translate to preventative treatment of colitis-associated colorectal cancer. The link between ulcerative colitis and colorectal cancer is widely accepted, however the extent of the risk is difficult to determine as reports describe widely varying rates (Eaden et al., 2001; Lakatos and Lakatos, 2008). However, the severity of colitis and duration of inflammation are contributing risk factors. Therefore, reducing either of these by targeting IL-13 could consequently reduce the risk of cancer. Indeed, Schiechl and colleagues provide strong evidence to validate this preventative approach (Schiechl et al., 2011) as blocking IL-13 or depleting NKT cells reduced inflammation, tumor size and tumor number. In summary, new treatment approaches that specifically target IL-13 and differentiate IL-4 from IL-13 signaling mechanisms could be key in developing a successful treatment strategy in a subset of patients identified with a specific biomarker, for example increased mucosal IL-13.

## Author contributions

The author confirms being the sole contributor of this work and has approved it for publication.

### Conflict of interest statement

The author declares that the research was conducted in the absence of any commercial or financial relationships that could be construed as a potential conflict of interest.
